# Investigation of Relative Development and Reproductivity Fitness Cost in Three Insecticide-Resistant Strains of *Aedes aegypti* from Thailand

**DOI:** 10.3390/insects10090265

**Published:** 2019-08-22

**Authors:** Jassada Saingamsook, Jintana Yanola, Nongkran Lumjuan, Catherine Walton, Pradya Somboon

**Affiliations:** 1 Center of Insect Vector Study, Department of Parasitology, Faculty of Medicine, Chiang Mai University, Chiang Mai 50200, Thailand; 2 Graduate PhD’s Degree Program in Parasitology, Faculty of Medicine, Chiang Mai University, Chiang Mai 50200, Thailand; 3 Department of Medical Technology, Faculty of Associated Medical Sciences, Chiang Mai University, Chiang Mai 50200, Thailand; 4 Research Institute for Health Sciences, Chiang Mai University, Chiang Mai 50200, Thailand; 5 School of Earth and Environmental Sciences, Faculty of Science and Engineering, University of Manchester, Manchester M13 9PL, UK

**Keywords:** *Aedes aegypti*, *kdr*, insecticide resistance, fitness

## Abstract

Knockdown resistance (*kdr*) and detoxification enzymes are major resistance mechanisms in insecticide-resistant *Aedes aegypti* throughout the world. Persistence of the resistance phenotype is associated with high fitness of resistance alleles in the absence of insecticide pressure. This study determined the relative fitness cost of three insecticide-resistant strains of *Aedes aegypti*—PMD, PMD-R, and UPK-R—and a hybrid under similar laboratory conditions in the absence of insecticide. The PMD strain is resistant to DDT with no *kdr* alleles; the PMD-R is resistant to DDT and permethrin with 1534C homozygous *kdr* alleles; and UPK-R is resistant to DDT, permethrin, and deltamethrin with 989P + 1016G homozygous alleles. The DDT-resistant PMD strain had the highest fitness compared with the two DDT/pyrethroid-resistant strains (PMD-R and UPK-R) and hybrid. Consistent fitness costs were observed in the DDT/pyrethroid-resistant strains and hybrid, including shorter wing length, reduced egg hatchability, shorter female lifespan, and shorter viability of eggs after storage, whereas no effect was observed on blood feeding rate. In addition, reduced egg production was observed in the PMD-R strain and prolonged developmental time was seen in the UPK-R strain. The corresponding hybrid that is heterozygous for *kdr* alleles was fitter than either of the homozygous mutant genotypes. This is in accordance with the high frequency of heterozygous genotypes observed in natural populations of *Ae. aegypti* in Chiang Mai city.

## 1. Introduction

*Aedes aegypti* is the primary mosquito vector of viral diseases such as dengue fever, yellow fever, chikungunya, and Zika, which are significant public health problems in many countries in tropical and sub-tropical regions [1,2,3]. In Thailand, the first epidemic of dengue and dengue hemorrhagic fever occurred in 1958. In 2018 alone, there were 85,849 dengue cases and 111 deaths reported in the country [4]. Several human candidate vaccines against dengue are still under development and there is no specific treatment. Further, strategies involving the release of either *Wolbachia* infected mosquitoes to decrease vectorial capacity [5], or genetically modified mosquitoes that suppress the natural populations [6,7], are currently being evaluated in the field and not available for large-scale applications. The control of disease transmission is, therefore, based mainly on the management of breeding sites and insecticide applications [8]. Organochlorines (DDT), organophosphates (e.g., malathion, fenitrothion, and temephos), and carbamates (e.g., propoxur) were extensively used for controlling mosquitoes for over 50 years in Thailand until pyrethroids were introduced in the early 1990s [9]. Pyrethroid insecticides have become the most popular insecticide because of their fast action coupled with low mammalian toxicity and lower environmental risk [10]. Pyrethroids and DDT act on the nervous system by modifying the gating kinetics of voltage-gated sodium channels (VGSC), resulting in rapid immobilization (knockdown) and death [11]. Several formulations of pyrethroids have been widely used for controlling adult *Ae. aegypti*, for both domestic (i.e., aerosol cans, mosquito coils, and impregnated materials) and public uses (i.e., thermal fogging and ultra-low volume (ULV) space spraying). The intensive and extensive use of insecticides has generated high selective pressure on mosquito populations and led to the development of insecticide resistance [1,8]. This problem is the principal factor reducing the efficacy of disease vector control programs.

In Thailand, DDT-resistant *Ae. aegypti* were first observed in the mid-1960s [12]. Nowadays, resistance to pyrethroids (permethrin and deltamethrin) and other classes of insecticides has been found throughout Thailand and other countries [13,14,15,16,17,18]. Insecticide resistance in *Ae. aegypti* consists of four mechanisms: increased metabolic activity of detoxifying enzymes [19,20], target site insensitivity or knockdown resistance (*kdr*) [21,22], cuticle thickening [23,24], and behavioral avoidance [9]. Metabolic resistance and *kdr* are considered to be of particular importance and well documented. Metabolic resistance is caused by elevated activity of enzymes via overexpression or conformational change of enzymes involved in the processes of insecticide detoxification. Three major classes of enzymes—mixed-function oxidases (MFO), esterases, and glutathione-S-transferases (GST)—have been reported to be involved in metabolic resistance to organochlorines, pyrethroids, and organophosphates [21]. *Kdr* is related to either single or multiple non-synonymous mutations in genes encoding VGSC protein, making it less susceptible to the binding of pyrethroids and DDT [22]. The resistance of *Ae. aegypti* in Thailand to pyrethroids is associated with *kdr* mutations [25,26] and, to a lesser extent, elevated activity of MFO enzymes, whereas esterases and GST play a little role [27,28,29,30]. However, little has been done to characterize metabolic resistance mechanisms using genome-wide transcriptional analyses and functional validations.

In Thailand and many other countries in Asia, three *kdr* mutations are common. The first is V1016G, a valine to glycine substitution at position 1016 within domain II of VGSC. It is associated with varying degrees of resistance to type I and II pyrethroids, such as permethrin and deltamethrin, respectively [25,31]. The 1016G allele has been found throughout Thailand, with an average frequency of 0.23 [25]. The second mutation is S989P, a serine to proline transversion at position 989, often found with V1016G [23,25,32,33]. The third is F1534C, a phenylalanine to cysteine substitution at position 1534 within domain III and is associated with resistance to type I pyrethroids [26]. In Thailand, the average frequency of the 1534C allele was 0.77 [34]. In Latin America, V1016I, a valine to isoleucine substitution, and F1534C are common [35,36]. Although *kdr* alleles are primarily recessive, heterozygous 989S/P + 1016V/G + 1534F/C mosquitoes are highly resistant to pyrethroids [28,37].

Although resistance mechanisms help mosquitoes survive under continuous insecticide pressure, these actions are costly and may negatively affect mosquito’s fitness (e.g., size of body, adult longevity, larval development time, fecundity, fertility, mating competitiveness, and blood feeding capability) when the insecticides are withdrawn. The fitness reduction may be caused by pleiotropy in the resistance genes themselves or as a consequence of hitch-hiking effects. Several studies have shown that insecticide resistance reduces the reproductive fitness of *Ae. aegypti* [38,39,40,41,42,43] as well as some *Anopheles* and *Culex* species [41,44,45,46,47,48,49]. For example, decreased longevity and increased larval development time have been reported in pyrethroid-resistant *Ae. aegypti* and also *Culex pipiens* [42,47,50,51]. In competition analyses, *Ae. aegypti* with *kdr* mutations had longer larval development times when reared alongside the susceptible strain under stringent conditions [39]. By contrast, pyrethroid-resistant *An. funestus* in southern Africa did not show any loss of fitness under laboratory conditions [52]. These studies have suggested that fitness cost may vary depending on mosquito species and resistance mechanisms. 

While investigation of fitness cost in field populations is difficult and complicated, evaluation of relative fitness obtained from laboratory studies is useful when considering the impact of resistance alone on biological fitness [39,42]. Over 20 years of our studies regarding insecticide resistance mechanisms in *Ae. aegypti* populations in Thailand, we have detected four major genotypic forms of *kdr* mutant mosquitoes; no *kdr* alleles (wild type), 1534C homozygote, 989P + 1016G homozygote, and 989S/P + 1016V/G + 1534F/C heterozygote [25,26,37]. We also succeeded in establishing three strains of *Ae. aegypti* harboring no *kdr* alleles (PMD strain), 1534C homozygous allele (PMD-R strain), and 989P + 1016G homozygous alleles (UPK-R strain). These strains also showed differences in levels of detoxifying enzyme activities and response to insecticide susceptibility [27,28,29,30,37,53,54,55,56]. It is known that maintenance of resistance including *kdr* alleles often involves a fitness cost in the absence of insecticide [39]. Recent studies revealed that the heterozygous form was most common (about 46% of population) in Chiang Mai Province, and showed a high level of pyrethroid resistance [28,37]. It was also postulated that the heterozygote might be fitter than homozygous mutant genotypes and plays an important role in maintaining 1534C and 1016G alleles in wild populations [37]. However, the maintenance of polymorphism for heterozygous alleles is not clearly understood. Herein, we compared several life-history parameters in order to evaluate the relative fitness cost in the three insecticide-resistant strains. Understanding these aspects would improve our knowledge on the impact of resistance alleles on fitness cost and help predict resistance trends in the future. If there is a fitness cost, it is possible to withdraw pyrethroids for a period to restore susceptibility that can be a useful tool in insecticide resistance management. 

## 2. Materials and Methods

### 2.1. Mosquito Strains

Three strains of *Ae. aegypti*—PMD, PMD-R, and UPK-R—as described above were used in this study. Initially, exposure of natural *Ae. aegypti* adults reared from wild larvae in northern Thailand to WHO DDT papers (4%) for 30 min revealed low or no mortality, indicating that DDT resistance was very common [13]. Attempts have been made to establish a DDT-susceptible strain but have not been successful. The PMD and PMD-R strains were established from isofemale lines reared from wild larvae from Ban Pang Mai Daeng, a rural village of Chiang Mai Province since 1997 [29]. Briefly, half of each isoline was exposed to WHO permethrin papers (0.25%) for 1 h. Isolines that showed 100% mortality were checked for *kdr* alleles by DNA sequencing and those without *kdr* alleles were pooled and colonized as the DDT-resistant or the pyrethroid-susceptible PMD strain. Exposure of female and male progeny to DDT papers continued for at least 10 generations. This strain is susceptible to permethrin and deltamethrin, but resistant to DDT (<5% mortality) [55].

The permethrin-resistant PMD-R strain was established from several 1534C homozygous isofemale lines that had survived permethrin exposure bioassays. Exposure of female and male progeny to permethrin papers continued for at least 10 generations. This strain is resistant to permethrin and DDT determined by adult bioassays (0–5% mortality), conferred by *kdr* and metabolic enzymes. DDT resistance in the PMD and PMD-R strains is associated with increased DDTase and MFO activities which were about 10-fold and 4-fold, respectively, higher than the susceptible Rockefeller strain [29]. Later, Prapanthadara et al. [53] revealed that DDTase activity of GST isoenzymes in the PMD-R was about 10-fold higher than the PMD strain. Although the amount of MFO in the PMD and PMD-R strains was not significantly different [30], there are, however, differences in another oxidative enzyme system between the two strains; two aldehyde dehydrogenase (ALDH) genes, ALDH9948 and ALDH14080 were upregulated in the PMD-R strain and involved in permethrin metabolism [27]. Mosquitoes of the 82nd generation of both strains were used in this study. 

The deltamethrin-resistant UPK-R strain was established from isofemale lines reared from wild larvae from Chiang Mai city since 2006 [28]. Several homozygous 989P + 1016G isofemale lines that had survived deltamethrin (0.05%) exposure bioassays were pooled and colonized. Exposure of female and male progeny to deltamethrin papers continued for at least 10 generations. This strain is resistant to not only deltamethrin (3–8% mortality), but also DDT and permethrin (0–5% mortality), conferred by *kdr* and metabolic enzymes. The UPK-R strain showed a greater level of MFO, esterase, and GST activities (1–2 fold) than the PMD strain [55], but DDTase activity has not yet been investigated. Mosquitoes of the 41st generation of this strain were used in this study. 

Our previous studies revealed that permethrin resistance levels of UPK-R and PMD-R were higher than the pyrethroid-susceptible PMD strain by 325-fold and 25-fold, respectively, as determined by larval bioassays, while their deltamethrin resistance levels were higher than PMD by 53-fold and 13-fold, respectively. The adults of PMD-R and UPK-R colonies were occasionally (1–2 times a year) exposed to the standard WHO permethrin (0.75%) and deltamethrin (0.05%) papers, respectively, to confirm maintenance of resistance status. No serious deteriorative signs of inbreeding depression, e.g., high mortality of immature stages and reduced longevity of adults, were observed in the three strains.

We created a heterozygote, harboring 989S/P + 1016V/G + 1534F/C alleles, by crossing UPK-R females with PMD-R males. The F_1_ hybrid showed intermediate resistance to permethrin and deltamethrin [28]. The genotypes of the three strains and the hybrid were confirmed by multiplex PCR and DNA sequencing [57]. 

### 2.2. Mosquito Rearing

All laboratory *Ae. aegypti* strains were reared and maintained in the insectary at 25–27 °C, ~70% relative humidity with a 12 h day/night cycle under our standard rearing conditions [25]. Dried mosquito eggs were placed into 25 × 35 × 6 cm-plastic trays filled with 3 L of distilled water for hatching. After hatching, the first instar larvae (approximately 300 per tray) were fed on finely ground dog biscuit (Tesco, Thailand). The water in the trays was changed two to three times a week to avoid stagnation. The pupae were transferred to plastic cups containing distilled water and placed into a 30 × 30 × 30 cm mosquito cage. After emergence, the adults were provided with 10% sucrose and 10% v/v multivitamin syrup (Seven Seas, Thailand) soaked onto cotton wool which was changed daily. One week after emergence, adult female mosquitoes were fed by an artificial membrane feeding method with heparinized cow blood [58]. Three days post feeding, an oviposition cup lined with filter paper and filled with distilled water was placed in the cage and females were allowed to lay eggs for a few days. The filter papers with eggs were air dried for 4–5 days and stored in a sealed plastic bag.

### 2.3. Effect of Fitness Cost on Biological Parameters 

#### 2.3.1. Developmental Time and Viability of Mosquitoes

This experiment was to compare the development time and viability of larvae until adult emergence in different strains under the same conditions. Eggs were submerged in water and the larvae hatched within 24 h were used. Four replicates of 50 larvae per strain were gently transferred to plastic cups (770 mL) containing 500 mL of distilled water. Thereafter, 50 mg of finely ground dog biscuit was offered daily until pupation. The numbers of pupae were counted daily until the last larva pupated. The numbers of adults emerged were counted and sexed. 

#### 2.3.2. Size of Mosquitoes

This experiment was to determine if there was a significant difference in the size of mosquitoes from different strains. The size of individual adult females was estimated by the average length of both right and left wings. Thirty females per strain obtained from the same rearing conditions from Section 2.3.1 were randomly selected. The wings were cut and placed on a glass slide with normal saline and covered by a coverslip. Wing length was measured as the distance from the axillary incision to the wing tip (the end of vein R_3_ [59]), using a digital camera attached to a compound microscope (BX53, Olympus, Japan) and cellSens standard software version 1.18 (Olympus, Tokyo, Japan).

#### 2.3.3. Blood Feeding Capacity

This experiment was to compare the amount of blood feeding by females of different strains. Seven-day-old females were deprived of sugar solution 24 h prior to blood feeding. After fasting, female mosquitoes were divided at random into two groups. The first group, triplicates of 100 females each, was transferred to a cage containing the artificial membrane feeder as above and allowed to feed for 30 min. The number of fully engorged females was counted. Shortly after feeding, they were killed by freezing in −20 °C for five minutes and weighed in pools (five each). The second group, without blood feeding, was killed and weighed in the same manner. The amount of blood taken was calculated from the ratio of female groups with and without a blood feeding. 

#### 2.3.4. Fecundity

This experiment aimed to determine if there was a significant difference in fecundity of females of different strains. Two hundred pairs of one-day-old males and females were confined in a cage for a week before a blood meal was offered as described above. Forty fully engorged females were randomly aspirated and kept individually in oviposition cups (237 mL) lined inside with filter paper and provided with a 10% sugar solution. Five days post feeding, 25 mL of distilled water was poured into the cup, and the females were allowed to lay eggs for three days. Deposited eggs were air dried and counted.

#### 2.3.5. Egg Viability after Storage

This experiment was to determine the viability of eggs after different storage durations. About 160 blood-fed females from Section 2.3.4 were allowed to lay eggs for three days in the cage with an oviposition cup lined with filter paper. The papers with eggs were air dried for a week for embryo development. Afterward, the papers were cut into small pieces (each piece containing at least 100 eggs, estimated visually) and stored in a tight plastic food container. Hatchability of eggs was determined on week 0, as the control, and every two weeks for 38 weeks. To determine egg hatchability, three pieces of papers were randomly picked and checked for the presence of lids before they were submerged for 24 h in 300 mL of distilled water mixed with 0.3 g of finely ground dog food to induce hatching. The egg hatching rate was determined by examining 100 eggs/paper under a stereomicroscope. Eggs with opened lids and no larva inside were counted as viable (successful hatching).

#### 2.3.6. Adult Longevity

This experiment was to determine if there was a significant difference in the longevity of adult mosquitoes among the different strains. Fifty pairs of males and females, one-day-old, were released in duplicate into 30 × 30 × 30 cm cages and provided with a 10% sugar solution which was changed daily. The bottom of the cages was lined with white paper so that dead mosquitoes were easily seen. The dead mosquitoes were counted and removed every day. Comparisons of survival curves were analyzed with both the log-rank (Mantel–Cox) test and Gehan–Breslow–Wilcoxon test using GraphPad Prism version 8.0.2 for Windows (GraphPad Software, San Diego, CA, USA).

#### 2.3.7. Larval Competition

This experiment aimed to determine the competitive ability between larvae of the PMD-R and UPK-R strains when they were reared together under conditions of limited room and food. Duplicates of 50 one-day-old larvae of each strain were put together in a small cup (237 mL) containing 100 mL distilled water. Then, 20 mg of dog food was added to each cup every other day. Pupae were separated daily and transferred to a new cup containing distilled water for the emergence of adults. The emerging adults were recorded daily and genotyped for 1016G and 1534C alleles by multiplex PCR [57]. 

#### 2.3.8. Colony Competition

This experiment was to determine the reproductivity of adults of the PMD-R and UPK-R strains when they were mixed together in the cages in the absence of insecticide pressure. To ensure that the females were virgin before releasing into the cages, pupae of each strain were sexed and separated. Forty one-day-old females of each strain were released, followed by forty males of both strains (80 pairs per cage in total). After one week, a blood meal was offered. Four days after blood feeding, an oviposition cup was introduced in the cage and allowed to stand for three days for egg laying. The F_1_ progeny eggs were air dried, induced to hatch, and reared to the adult stage under the standard laboratory conditions as above. Thirty adult females and males in each cage were randomly selected and genotyped. Since our previous study revealed that the 989S/P allele was found in all the PMD-R × UPK-R F_1_ hybrids, we determined only the frequency of 1016G and 1534C alleles by multiplex PCR [57]. 

### 2.4. Statistical Analysis

Comparisons of mean values of life-trait parameters among PMD, PMD-R, UPK-R, and F_1_ (PMD-R × UPK-R) were conducted using analysis of variance (ANOVA), followed by Bonferroni’s multiple comparison test. Qualitative data were analyzed by Fisher’s exact test. Others were indicated in experimental methods or results. Analyses were made using Prism version 8.0.2 for Windows (GraphPad Software, San Diego, CA, USA).

## 3. Results

### 3.1. Development and Fecundity 

**Figure 1:**
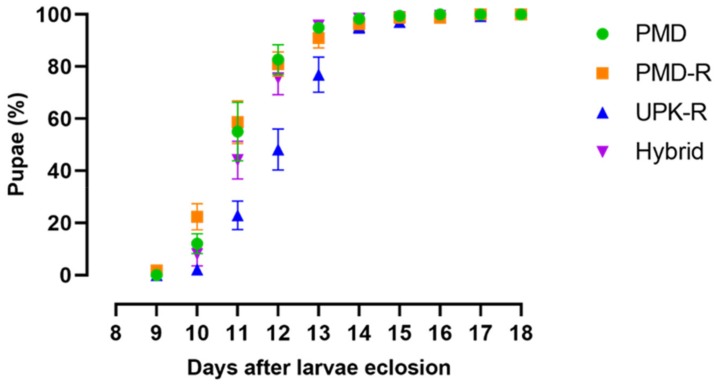
Development time of larvae to pupae of PMD, PMD-R, UPK-R strains, and F_1_ (PMD-R × UPK-R) hybrid. The cumulative mean percentage of pupae formation and standard error are indicated.

The mean duration of larvae developing to pupae in the deltamethrin-resistant UPK-R strain was about one day longer than the other strains and hybrid, while the pupation and adult emergence rates were not significantly different. Consequently, the adult emergence time in the UPK-R strain was about one day longer than the others, as shown in Table 1 and Figure 1. The observed sex ratio was 1:1 for all, as shown in Table S1. Significant differences in mean wing length were observed; specimens from the pyrethroid-susceptible PMD strain had the longest wing lengths, followed by UPK-R and hybrid mosquitoes, whereas those from the permethrin-resistance PMD-R strain were the shortest. Mean numbers of eggs per female were not significantly different, except for PMD-R females which laid about 15–20% fewer eggs than the others. The mean egg hatching rate of the PMD strain was about 12–16% higher than the other strains and hybrid. The hatching rate of hybrid eggs was about 5% lower than the two parental resistant strains. Therefore, the biological fitness of the pyrethroid-susceptible PMD strain (no *kdr* alleles) was greatest, followed by the hybrid which appeared superior to the pyrethroid-resistant PMD-R (1534C homozygote) and UPK-R (989P + 1016G homozygote) strains.

### 3.2. Blood Feeding Capability

Blood feeding rates among the three strains and hybrid were not significantly different, as shown in Table 2. The average weights of pyrethroid-susceptible PMD and hybrid specimens, measured in pools, were greater than those from the pyrethroid-resistant PMD-R and UPK-R strains. However, the amount of blood ingested by PMD specimens was significantly greater than the others. The increasing weight ratios after blood feeding were not significantly different in all groups, indicating that their capacity of blood ingestion was similar regardless of body size.

### 3.3. Viability of Eggs after Storage

**Figure 2:**
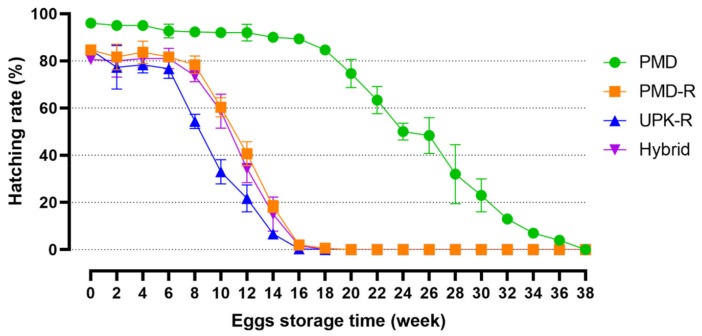
Egg hatching rates of PMD, PMD-R, UPK-R, and F_1_ (PMD-R × UPK-R) hybrid. Mean and standard deviation are indicated.

The hatching rate of pyrethroid-susceptible PMD eggs was significantly higher than the others, as shown in Figure 2, with about 50% hatchability after storage for 24 weeks. By contrast, the hatching rate of UPK-R eggs was reduced by 50% after 9 weeks. The hatching rates of PMD-R and hybrid eggs were not significantly different, with about 50% hatchability after 11 weeks.

### 3.4. Adult Longevity

**Figure 3:**
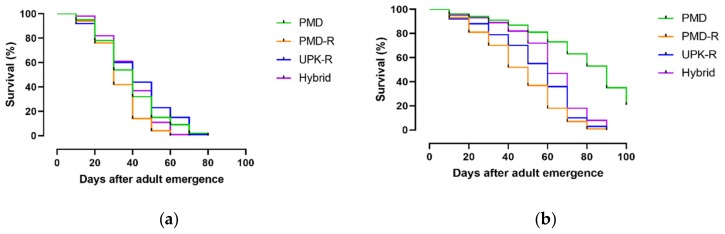
Survival curves of males (**a**) and females (**b**) of PMD, PMD-R, and UPK-R strains, and F_1_ (PMD-R × UPK-R) hybrid under laboratory conditions.

Longevity of males was generally less than the females, as shown in Figure 3a,b. The longevity of males from the PMD-R strain was the shortest (medium survival time, MST 30 days) whereas the rest were not significantly different (MST 40 days), as shown in Table S2. The longevity of pyrethroid-susceptible PMD females was longest (MST 90 days), whereas the PMD-R was shortest (MST 50 days). No significant differences were observed between the female UPK-R and F_1_ (PMD-R × UPK-R) hybrid (MST 60 days), as shown in Table S2. 

The relative fitness cost on life-trait parameters of the pyrethroid-resistant strains and the hybrid, compared with the pyrethroid-susceptible PMD strain, is summarized in Table 3. The resistant strains showed deteriorative effects on six out of eight life-trait parameters, whereas the hybrid showed only four, indicating that the hybrid had higher fitness than its parents.

### 3.5. PMD-R and UPK-R Competition 

#### 3.5.1. Larval Competition

**Figure 4:**
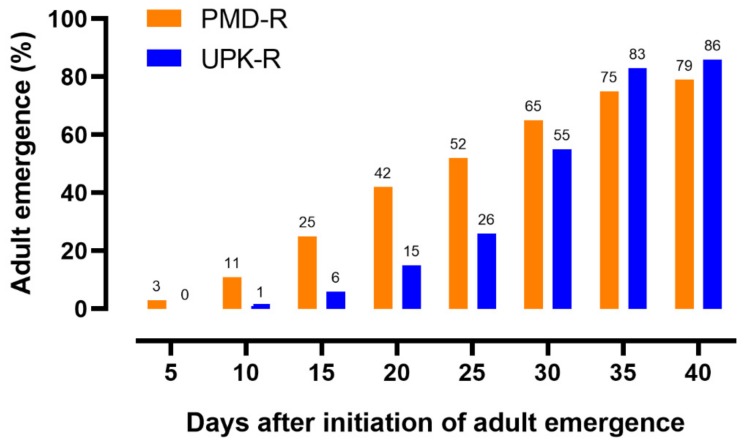
Cumulative adult emergence from the first appearance until the last emergence of PMD-R and UPK-R strains when they were reared together under limited space and food. The percentages of emerging adults are indicated above each bar.

When larvae from the PMD-R and UPK-R strains were reared together under limited space and food, larval development was prolonged, as shown in Figure 4. About 50% cumulative adult emergence of PMD-R took place on day 25 which was about 5 days faster than UPK-R. However, the final emergence rate of UPK-R adults was higher than PMD-R by 7%.

#### 3.5.2. Colony Competition

When forty pairs of males and females of PMD-R (homozygous 1534C/C allele) and UPK-R (homozygous 989P/P + 1016G/G) strains were confined in the same cages, the F_1_ adult progeny reared from oviposited eggs showed that the heterozygous (1016V/G + 1534F/C) genotype predominated (46.7%) (P allele not determined), followed by homozygous 1016G/G + 1534F/F (38.3%) and homozygous 1016V/V + 1534C/C (15.0%), as shown in Table 4. The calculated G allele and C allele were 0.63 and 0.37, respectively.

## 4. Discussion

The current study determined relative fitness of a pyrethroid-susceptible strain (PMD) and two pyrethroid-resistant strains (PMD-R and UPK-R), including the F_1_ hybrid (PMD-R × UPK-R) of *Ae. aegypti* under similar laboratory conditions. Unfortunately, *Ae. aegypti* populations in Thailand are widely resistant to DDT [13] and hence, a fully susceptible strain was unavailable for comparison. Pyrethroid resistance in our resistant strains is conferred by *kdr* mutations and, to a lesser extent, metabolic enzymes (MFO) based on a synergist assay [28]. In addition, since all strains originated from Chiang Mai province, and were resistant to DDT, their genetic backgrounds are expected to be genetically similar to each other and to the natural populations on which they were based. This allows us to reasonably infer that the major contributor to relative fitness differences in this study depends on *kdr* genotype and that the responses found are likely to be relevant to the natural populations. 

The pyrethroid-susceptible PMD strain had the highest fitness compared with the two pyrethroid-resistant strains (PMD-R and UPK-R) and the hybrid. The resistant strains showed fitness costs on six out of eight parameters, as shown in Table 3, whereas only four parameters were affected in the hybrid. The resistance ability of the hybrid is intermediate between the two resistant strains [28], suggesting that the fitness cost is positively related to the level of resistance. The fitness parameters that were affected in all the homozygous and heterozygous *kdr* genotypes were shorter wing length, reduced egg hatchability, shorter female lifespan, and decreased viability of eggs after storage, whereas no effect was observed on blood feeding rate. The effects on developmental time, egg production, and blood ingestion were variable among *kdr* genotypes. 

The UPK-R strain, which displayed the highest pyrethroid resistance level, had significantly prolonged larval development times, resulting in increased adult emergence times compared to the other genotypes (PMD, PMD-R, and hybrid). This indicates that the 989P + 1016G *kdr* alleles may be particularly detrimental. In *An. gambiae*, Platt et al. [60] reported an additive fitness cost in male homozygous mutations for both sodium-gated (*kdr*) and GABA-gated chloride channels. 

The prolonged larval development time in the UPK-R strain was confirmed when the larvae of this strain were reared together with PMD-R larvae under limited space and food. This result agrees with other previous studies showing that increased development time occurred in *Ae. aegypti* and *Cx. quinquefasciatus* selected for pyrethroid resistance in the laboratory [39,42,47] and also in field collected *Ae. aegypti* with high resistance to organophosphates [42]. Prolonged larval development is disadvantageous as it increases the risk of exposure to extrinsic factors such as container habitat elimination, predation, disease, or xenobiotics, all of which can reduce larval survival [61,62] and will likely also involve a competitive disadvantage for resources. Shorter development time can therefore boost the chance of adult emergence, increasing vector density which is one aspect of vectorial capacity. Development thresholds could explain the delay in developmental time. Larvae require a certain amount of accumulated nutrients to trigger metamorphosis to the next stage. Resistant larvae may need more resources to sustain resistance mechanisms, leading to increased development thresholds [40]. However, Deniz et al. [40] suggested that prolonged development times might benefit temephos-resistant *Ae. aegypti*, allowing for better use of nutrients available in the rearing container which could ease or compensate for the fitness disadvantage of maintaining resistance mechanisms. 

Wing length indirectly represents the body size of adult mosquitoes. Specimens from the pyrethroid-susceptible PMD strain were bigger than those of the resistant strains and hybrid, suggesting that resistance alleles had an adverse effect on the size of mosquitoes, as found in our previous study [63]. Furthermore, among the resistant strains (PMD-R and UPK-R), their body sizes were significantly different from each other, suggesting that the degree of size reduction was dependent on the level of resistance or different *kdr* alleles. The relationship between temephos resistance and short wing length in *Ae. aegypti* has also been reported elsewhere [40]. However, Jaramillo et al. [51] found that the resistance affected only the wing shape but not the wing size in *Ae. aegypti* resistant to lambda-cyhalothrin. In *Culex* mosquitoes, shorter wing lengths were seen in *Cx. pipiens* that overproduced acetylcholinesterase [61]. Additionally, pyrethroid-resistant *Cx. quinquefasciatus* mosquitoes with elevated levels of P450 enzymes were smaller in size than those of the susceptible strain [45]. 

Blood feeding capacity is an essential parameter for determining vectorial capacity since parasite or pathogen loads and number of eggs produced are directly proportional to the volume of ingested blood. Blood feeding rates in all groups were similar, ranging from 93.0% to 95.7%, and indicated that the resistance alleles did not affect the ability to respond to blood meal stimuli. In contrast, a substantial reduction of feeding rate was observed in deltamethrin and temephos-resistant *Ae. aegypti* females under laboratory conditions [38]. Resistant female mosquitoes are likely to ingest a lower blood volume compared to susceptible individuals [38,42,47,48]. Due to smaller body size, the volume of blood ingested by the two resistant strains was less than the susceptible PMD strain. Mosquito weights taken before a blood meal can be used to infer body size and wing length, and the results for both parameters were correlated as we expected. However, the weight increase ratios were not different, indicating no fitness cost on blood feeding capacity in the resistant strains. This implies that reduced blood ingestion was related to the difference in mosquito body size, not to the reduced willingness of blood sucking.

Reduced egg production and egg hatchability in insecticide-resistant *Ae. aegypti* has been reported elsewhere [41,42,43]. In *An. funestus*, however, egg production rates between the pyrethroid-resistant and susceptible strains did not vary by much [52]. In the current study, only the PMD-R females laid fewer eggs, suggesting that not all resistance alleles affect egg production. The resistance alleles are correlated with reduced egg hatchability (by 12–16%). Thus, reduced larval production would lead to a reduction of adult density and a decrease in vectorial capacity among resistant mosquitoes. 

The eggs of *Ae. aegypti* can tolerate dry conditions for a very long time [64]. They are ready to hatch once submerged in water. This property is an advantage for their survival under periods of desiccation. Moreover, the eggs of this species are the main form of passive dispersal, making it widespread and difficult to control. Under continuous insecticide exposure, this ability is very crucial for eggs of resistant mosquitoes because it helps to maintain resistant individuals (as well as resistant genes or mechanisms) in nature. In the current study, PMD eggs showed longer viability than the others after storage in dry conditions, whereas the UPK-R showed the most rapid loss of viability. About 90% of PMD eggs survived after 18 weeks of storage, but none of the eggs from the resistant strains or hybrid survived. This suggests that the survival of eggs under desiccation is shorter when the resistance level is increased. This fitness cost has an impact on the maintenance of resistant alleles during periods of prolonged desiccation. The viability rate of PMD, which is resistant to DDT, at week 21 was about 70% which is similar to the egg viability rate of all temephos-resistant groups of *Ae. aegypti* at day 150 (80%) reported by Diniz et al. [40]. However, results under laboratory conditions may not reflect what would happen in nature. Therefore, the viability of eggs of resistant mosquitoes under natural conditions needs further investigation.

Our results clearly demonstrated that the longevity of *Ae. aegypti* adults was reduced in the resistant strains. Over 80% of the PMD females survived up to 60 days which was slightly longer than other studies [38,42]. The longevity of PMD-R mosquitoes, despite having lower resistance, was shorter than UPK-R mosquitoes of both sexes. Large variations in longevity of resistant mosquitoes have been observed in previous studies. Reduced longevity has been observed in pyrethroid-resistant *Ae. aegypti* and *Cx. pipiens pallens* [42,47,51], and also temephos-resistant *Ae. aegypti* [38,40]. By contrast, Hardstone et al. [45] reported that *Cx. quinquefasciatus* females resistant to permethrin survived longer than those of susceptible strain when provided with sugar. However, no differences in longevity were observed in some studies, for example, between pyrethroid-resistant (Rock-*kdr*) and susceptible (Rock) *Ae. aegypti* strains [39], and also between some strains of *An. gambiae* and *An. stephensi* which were resistant to gamma-HCH and dieldrin [49]. This implies that the longevity of resistant adults in the laboratory may depend on the resistance mechanism and/or associated genetic background. Longevity reduction in females is important since this would affect the extrinsic incubation period of pathogens as well as blood feeding frequency, both of which are major aspects of vectorial capacity. Survival in the laboratory, however, cannot reflect the longevity of wild *Ae. aegypti* mosquitoes which usually live for a few weeks [65]. Additionally, lab conditions may not reflect their reproductivity in nature since male and female mosquitoes are usually only reproductively active in the first few weeks after emergence [66]. Therefore, the longevity and reproductivity of resistant mosquitoes in natural conditions should be further investigated.

Previous studies among laboratory-reared *Ae. aegypti* showed that large males had greater mating capacities than small males [67,68,69]. Berticat et al. [70] performed a mating competition in *Cx. pipiens* mosquitoes which revealed that susceptible males had more mating competitiveness (with both susceptible and resistant females) than those resistant to organophosphate. In *An. gambiae*, *kdr* heterozygote males were more likely to mate than homozygote resistant males and were more competitive than homozygote susceptible males [60]. However, there was no fitness cost on mating and insemination capability in a mating competition experiment between a susceptible *Ae. aegypti* strain (Rock) and a pyrethroid-resistant (Rock-*kdr*) strain [39]. In the current study, when adults of UPK-R and PMD-R were confined in the same cage, the heterozygous form predominated in the F_1_ progeny, with a higher frequency of the 1016G allele over the 1534C allele. This may be explained by the result of the present study that showed that the UPK-R strain produced more eggs than the PMD-R strain, as shown in Table 1. In addition, the body size of UPK-R mosquitoes was larger than those of the PMD-R strain, and likely gave them an advantage in mating competition. Under natural conditions, however, it is not known if resistant *Ae. aegypti* mosquitoes harboring homozygous 989P + 1016G alleles have more mating capability than those with the homozygous 1534C allele. 

In Chiang Mai city, Plernsub et al. [37] reported that the majority (46%) of *kdr* genotypes in an *Ae. aegypti* population consisted of the heterozygous form, followed by the homozygous 1534C (39%) and homozygous 989P + 1016G (15%) forms. The frequency of these *kdr* alleles appears to have increased only very slowly over the last decade of monitoring [25,34]. This may be due to balancing selection under insecticide pressure in relation to the fitness cost of resistance alleles. Although the fitness costs of resistance among natural populations of mosquitoes remains to be determined, the results of the current study suggest that the fitness of life-trait parameters in the heterozygous form may be higher than the homozygous 989P + 1016G mutant (UPK-R), which had increased larval development times as well as decreased viability of eggs under desiccation. Fitness was also higher than that seen in the homozygous 1534C mutants (PMD-R), which had smaller body sizes, lower fecundity, and decreased longevity. Similarly, the fitness of heterozygote resistant males was greater than that of homozygotes in *An. gambiae* [60]. Since the heterozygous mutants expressed a high pyrethroid-resistant phenotype, this has a significant impact on *Ae. aegypti* control programs using pyrethroid-based approaches [28,37]. Therefore, there is a need for both alternative methods or chemicals that overcome *kdr* in the long term (such as insect growth regulators), as well as continued monitoring of resistance genotypes in *Ae. aegypti* populations.

## 5. Conclusions

Collectively the data presented here on Chiang Mai derived laboratory strains indicate that in the absence of pyrethroid insecticides *Ae. aegypti* mosquitoes in Chiang Mai that carry *kdr* alleles, particularly in the homozygous form, are likely to have reduced fitness compared to non-*kdr* genotypes. This fitness cost has a broad basis in multiple life history, development, and reproductive traits including prolonged development time, smaller body size, decreased blood ingestion amount, lower fecundity, reduced egg hatching rate, shorter lifespan, and decreased viability of eggs under desiccation. The high use of pyrethroid insecticides in Chiang Mai coupled with the high fitness of the *kdr* allele hybrid in both the absence and presence of insecticide are likely maintaining both *kdr* alleles in Chiang Mai. Despite this, the much higher fitness of non-*kdr* genotypes in the absence of insecticide means that it is theoretically possible to restore pyrethroid susceptibility to Chiang Mai populations by the periodic withdrawal of pyrethroid insecticides that would favor the spread of non-*kdr* alleles. Given the primary importance of pyrethroids for disease control and the continuing threat from arboviral diseases vectored by *Ae. aegypti*, the plausibility of this approach should be given consideration.

## Figures and Tables

**Table 1:** Comparison of life-trait parameters of PMD, PMD-R, and UPK-R strains and F_1_ (PMD-R × UPK-R) hybrid.

Parameter	Strain (Mean ± SE) *			
	PMD	PMD-R	UPK-R	Hybrid
Pupation time (day) ^1^	11.6 ± 0.1 ^a^	11.5 ± 0.1 ^a^	12.6 ± 0.1 ^b^	11.8 ± 0.1 ^a^
Pupation rate (%) ^2^	89.5 ± 4.0 ^a^	85.0 ± 2.5 ^a^	89.0 ± 1.3 ^a^	95.0 ± 1.7 ^a^
Adult emergence time (day) ^3^	14.9 ± 0.1 ^a^	14.6 ± 0.1 ^a^	15.8 ± 0.1 ^b^	14.9 ± 0.1 ^a^
Adult emergence rate (%) ^4^	89.0 ± 3.8 ^a^	85.0 ± 2.5 ^a^	87.5 ± 1.0 ^a^	95.0 ± 1.7 ^a^
Wing length (mm)	3.29 ± 0.02 ^a^	2.98 ± 0.03 ^b^	3.14 ± 0.02 ^c^	3.08 ± 0.01 ^c^
Eggs/female (egg)	94.8 ± 2.6 ^a^	74.7 ± 3.8 ^b^	91.9 ± 1.7 ^a^	87.4 ± 1.6 ^a^
Eggs hatching rate (%)	96.0 ± 0.6 ^a^	84.7 ± 0.3 ^b^	84.7 ± 0.3 ^b^	80.7 ± 0.9 ^c^

* Values indicate mean and standard error. The same superscript letters in each row indicate no significant difference (*p* > 0.05, ANOVA, followed by Bonferroni’s multiple comparison test). ^1^ Duration of L1 larvae developing to pupae. ^2^ Percent of larvae developing to pupae. ^3^ Duration of L1 larvae developing to adults. ^4^ Percent of larvae developing to adults.

**Table 2:** Blood feeding capacity of the PMD, PMD-R, UPK-R, and F_1_ (PMD-R × UPK-R) hybrid.

Parameter	PMD	PMD-R	UPK-R	Hybrid
Blood feeding rate (%)	94.0 ± 0.6 ^a^	93.0 ± 0.6 ^a^	95.0 ± 1.2 ^a^	95.7 ± 0.3 ^a^
No. of pool ^1^	6	6	6	7
Before blood meal (mg) ^2^	12.58 ± 0.23 ^a^	9.90 ± 0.61 ^b^	10.75 ± 0.12 ^b^	11.54 ± 0.31 ^a^
After blood meal (mg) ^2^	27.55 ± 0.45 ^a^	20.37 ± 0.73 ^b^	22.18 ± 0.28 ^b,c^	23.83 ± 0.43 ^c^
Weight of blood ingested (mg) ^2^	14.97 ± 0.25 ^a^	10.47 ± 0.76 ^b^	11.43 ± 0.31 ^b^	12.29 ± 0.43 ^b^
Weight increase ratio	2.19 ± 0.02 ^a^	2.09 ± 0.13 ^a^	2.07 ± 0.03 ^a^	2.07 ± 0.06 ^a^

Values indicate mean and standard error. The same superscript letters in each row indicate no significant difference (*p* > 0.05, ANOVA, followed by Bonferroni’s multiple comparison test). ^1^ Five females in each pool. ^2^ Average per pool.

**Table 3:** Summary of relative fitness cost on life-trait parameters of the pyrethroid-resistant PMD-R and UPK-R strains and F_1_ (PMD-R × UPK-R) hybrid compared with the pyrethroid-susceptible PMD strain.

Life-Trait Parameter	PMD-R	UPK-R	Hybrid
Developmental time (larval to adult)	equal	longer	equal
Wing length	shorter	shorter	shorter
Blood feeding rate	equal	equal	equal
Blood ingestion volume	smaller	smaller	equal
Eggs/female	lower	equal	equal
Egg hatchability	lower	lower	lower
Egg viability duration	shorter	shorter	shorter
Adult longevity	shorter	shorter	shorter

**Table 4:** Frequencies of V1016G and F1534C alleles in F_1_ adult progeny reared from eggs obtained from cages containing equal pairs of males and females of PMD-R and UPK-R strains.

*Kdr* Alleles	No. of Mosquito (%)		Total
	Cage		
	A	B	
1016V/V + 1534C/C	5 (16.7)	4 (13.4)	9 (15.0)
1016G/G + 1534F/F	10 (33.3)	13 (43.3)	23 (38.3)
1016V/G + 1534F/C	15 (50.0)	13 (43.3)	28 (46.7)
Total	30 (100.0)	30 (100.0)	60 (100.0)

## Supplementary Materials

The following are available online at https://www.mdpi.com/2075-4450/10/9/265/s1, Table S1. *Aedes aegypti* sex ratio of each strain, Table S2. *P* value of pair comparisons of survival curves of each strain.
