# Supplementary material for: Investigation of Relative Development and Reproductivity Fitness Cost in Three Insecticide-Resistant Strains of Aedes aegypti from Thailand
Source: Insects. 2019 Aug 22;10(9):265. doi: 10.3390/insects10090265 (PMC6780153; doi:10.3390/insects10090265)
Supplement: Supplementary file 1 [file insects-10-00265-s001.pdf]

insects

Supplementary Materials

Table 1. *Aedes aegypti* sex ratio of each strain.

Strain	Male	Female	*p*
PMD	102	76	0.2021
PMD-R	93	77	0.4472
UPK-R	84	91	0.7484
Hybrid	109	81	0.1810

Numbers of males and females obtained from PMD, PMD-R, UPK-R and hybrid strains. *p* = probability for Fisher's exact test.

Table 2. *P* value of pair comparisons of survival curves of each strain.

Comparison	sex	Log-rank (Mantel-Cox) Test			Gehan-Breslow-Wilcoxon Test		
		χ^2	df	*p*	χ^2	df	*p*
PMD vs PMD-R	Male	9.43	1	**0.0021**	4.79	1	0.0286
	Female	91.26	1	**<0.0001**	71.42	1	**<0.0001**
PMD vs UPK-R	Male	1.60	1	0.2054	1.11	1	0.2927
	Female	69.09	1	**<0.0001**	50.89	1	**<0.0001**
PMD vs Hybrid	Male	0.09	1	0.7605	0.44	1	0.5067
	Female	51.56	1	**<0.0001**	34.48	1	**<0.0001**
PMD-R vs UPK-R	Male	19.36	1	**<0.0001**	10.02	1	**0.0015**
	Female	7.55	1	**0.0060**	7.52	1	**0.0061**
PMD-R vs Hybrid	Male	12.29	1	**0.0005**	10.36	1	**0.0013**
	Female	26.03	1	**<0.0001**	27.08	1	**<0.0001**
UPK-R vs Hybrid	Male	3.98	1	0.0461	0.49	1	0.4851
	Female	5.87	1	0.0154	5.95	1	0.0147

Family-wise significance level *p* value = 0.05. Bonferroni corrected threshold *p* value = 0.0083. *P* value less than 0.0083 was considered a significant difference (bold letter).
